# Radiomics and Quantitative MDA Criteria in Breast Cancer with Bone Metastases by MRI: Examples of Calculation Algorithms and Their Practical Use

**DOI:** 10.17691/stm2024.16.3.01

**Published:** 2024-06-28

**Authors:** V. Steinhauer, G. Hartung

**Affiliations:** Dr.-Ing., Software Architect; Devoteam GmbH, Wiesenstraße 14D, 64331 Weiterstadt, Hessen, Germany; Prof. Dr. med., Oncologist, Hematologist; MVZ am Obertor GmbH, Wilhelm-Seipp-Straße 3, 64521 Groß-Gerau, Germany

**Keywords:** radiomics, neural networks, breast cancer, temporal sequences, spinal metastases, MDA criteria

## Abstract

In the practical work of a radiologist or oncologist, especially in the context of individualized treatment, there is a need for fast and in-depth diagnostics. Radiomics and artificial intelligence can be of great help here. Quantitative and qualitative assessments obtained in this way act as decision support for the physician.

**The aim of the study** is to enhance the ability of quantitative and qualitative assessment of metastatic spinal lesions in breast cancer, to better evaluate the nature of changes under the influence of therapy, in addition to MDA.

**Materials and Methods:**

We used MRI data in sagittal projection for a patient diagnosed with T2N3M1 breast cancer when treated according to accepted clinical protocols. Metastases to the spine were assessed by a radiologist and by machine analysis using the described software: image internal structure extraction operators and recognition based on traditional neural networks. Fragments of the program codes used are also given.

**Results:**

The structure of metastatically changed vertebrae in sagittal projection was analysed using machine operators of image analysis. Subtle changes in structure such as several types of “calderas” and the pattern of change in image complexity as treatment with CDK 4/6 inhibitors were detected. Measurements were supported by metastasis recognition using neural networks, to increase the reliability of the estimates. In addition to the ability to record response to therapy, a fundamental ability to assess the degree of action compared to previous therapy was identified.

**Conclusion:**

The study showed high efficiency of using image structure analysis algorithms, good correlation of the results obtained with the radiologist’s opinion and with clinical and laboratory data, and allowed to approach the analysis of subtle effects to obtain not only quantitative characteristics in addition to MDA, but also to obtain new qualitative results.

## Introduction

At present, progress in diagnostics is not least ensured by the development of computer tools. First of all, it is image processing, the possibilities of which are not fully utilized. Among the problems in the direction of image use are insufficient theoretical developments, namely: the relationship of image artifacts to medical artifacts, physical manifestations in tissues represented in images, normalization of images, lack of universalization of software plug-ins — hints for various DICOM Viewer and much more. Of course, it is impossible to cover all these topics in a single article. Therefore, we narrowed down the task to spinal metastases in breast cancer, analysis of their changes over time, differential diagnosis — neural network cues for quantitative assessment of MDA criteria and outlined the development of methods (Arcela, Caldera) presented in the paper [1], on MRI in sagittal projection. In [1] more information about these markers and their correlations with a common breast cancer marker such as CA 15-3 can also be found.

As our practice has shown, long-term changes are better tracked in the sagittal projection, and thus there is an opportunity to study the effect of drugs on markers more subtly, which allowed us to record the response to individual drugs (cyclin-dependent kinase 4/6 (CDK 4/6) inhibitors palbociclib, abemaciclib). The arsenal of depth image processing tools was chosen precisely for the detection of subtle morphological effects.

In doing so, in this paper we have added important aspects such as normalization and data control for training neural networks. In the most important cases, we provide not only parameters for using certain software packages, but also direct codes for quick verification by developers. The programming language in this paper is Java, easily portable and widely used.

## Materials and Methods

### Radiomica Applicata

The software used in this work includes both proprietary modules and modules of well-known libraries combined in DICOM MRT/CT Viewer. Programming in the vast majority of cases was carried out in the Java/JavaFX language, which provides high portability, sufficient speed and availability of a huge number of graphical and computational capabilities of this language. The experimental system is realized as a separately installable software module (Radiomica Applicata). To process DICOM data the dcm4che v5 program library is used [2]. The working panel has a classical structure: menu at the top, image tree on the left, images on the right. The control is as close as possible to what is known in the standard DICOM Viewer. The system has help in several languages.

### Structural markers and their temporal sequences

Time sequences are often used in radiology to track diseases. The goal of our application was to simplify and automate this process as much as possible. Therefore, with a few simple steps, curves of change of indicator markers with time are plotted. Unfortunately, the considered markers are not available in the literature, so it is not possible to make a comparison. For control we used expert evaluations and data of such common biochemical markers as CA 15-3 [1]. Spine MRI was used to illustrate temporal sequences. Temporal sequences are memorized and allow stability experiments with changes in image window, brightness, and so on. An important prerequisite is that the image is acquired in a single instrument mode. These issues are well covered in the recommended work [3]. Within these recommendations, we need to compare morphological features: biophysical process → morphological representation → quantification. For this purpose, we use intensity estimation by [4], which gave a possible contrast spread of about 5–6%. The linear correlation coefficient (Pearson’s coefficient) between DICOM RepetitionTime/EchoTime and the measured parameters is less than 0.3, indicating at least linear independence of the examples.

### Operator Arcela

**Figure 1.**
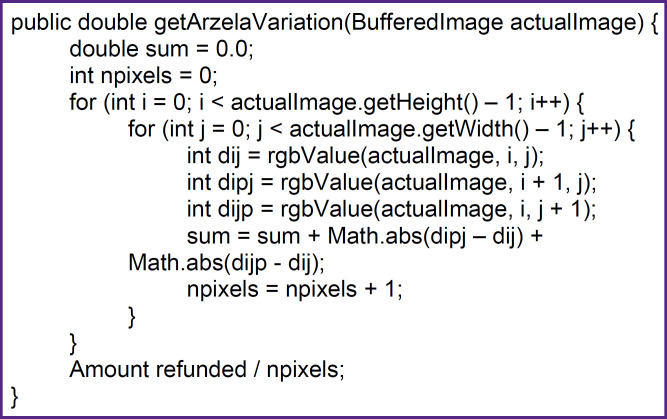
The calculation work function

The first operator we used is Arcela. Its detailed consideration, as well as the two following ones, can be found in reference [1]. The need for such a quantitative characterization arises in the first point of the MDA criteria, the numerical characterization of the total response. This operator is used to evaluate the complexity of an image: one image will be more complex than another if the sum of the boundaries of its constituent objects is larger. For example, inflammation around a tumor increases the sum of contrast intensity gradients observed as angiogenesis in pathological conditions, which makes the overall image more complex and thus shows the effect of therapy. Thus, to calculate the complexity value, each selected image segment taken in the temporal sequence is reduced in physical size to the minimum Pixel Spacing from the sequence. Since this is a black and white image, the unified BufferedImage.TYPE_BYTE_GRAY for Arcela is selected for gray scaling, which is performed in the next step. In this step, the RGB values of the pixels are brought to a unified view. The calculation work function is given in Figure 1.

Otherwise, the calculation of Arcela values has no other peculiarities. The best results were obtained when choosing a window covering the inner part of the sagittal slice of the vertebra, as well as in all subsequent markers.

### Caldera and its meanings

Proceeding from the fact that in breast cancer many objects in bone have radial symmetry, not only in the form of metastases, but also in the form of inflammatory foci and “sclerosing” zones induced by them, we set out to evaluate them quantitatively, that is, to numerically characterize the partial response, and, if possible, to classify them. For simplicity, we have grouped them under a common name based on their appearance, calderas. Their area on a vertebra in sagittal projection was taken as a quantitative characterization so that temporal sequences of change from similar objects could be plotted. Caldera values are expressed as a percentage of the selected window size (ROI), or, in other words, of the sagittal area of the selected vertebra. The system distinguishes three types of calderas according to their intrinsic intensity relative to the intensity of the intervertebral disc: Black (black, darker than the disc — indicated by green), White (lighter than the disc — indicated by yellow), ISO (approximately equal to the intensity of the disc — indicated by red). The latter, according to our observations, change frequently and abruptly. There are many reasons for this; accordingly, the explanation of ISO is problematic. White on T2 is of particular interest. With the beginning of therapy with one or another inhibitors CDK 4/6 their area increases, gradually coming to naught after about 6–10 months.

Here we use the package functions from [5]. The most important feature of our algorithm is the automatic selection of the threshold parameter by finding the maximum of the total area of ellipses of a given type (White, Black, ISO).

### Long-term changes in contrast accumulation

The use of contrast is widely known. Therefore, we propose to use the accumulation dependence as a marker of the disease process in this package as well. In this case, pre-interpolation using Bicubic Interpolation (InterpolationType.BICUBIC) before the Blur operation worked well. The threshold value was calculated as 0.9 of the average intensity of the acquired image. Contours of contrast (polygons) are calculated from the filtered (see Blur) grayscale image using the function from [5] BinaryImageOps.contour(filtered, ConnectRule.EIGHT, null).

### Features of recognizing metastases in the spine

In addition, the program complex allows differential analysis with the help of neural networks. Let’s consider one of the typical tasks: recognizing metastasis, normal tissue and hemangioma. For this purpose, we will apply MLP (Multilayer Perceptron). Pre-processing involves normalizing the data and checking it for separability, because a neural network is nothing but a classifier. We will take MRI T2 images of thoracic vertebrae as the images to be classified. To improve the reliability of classification, it is desirable to normalize the images for both the training array and the test objects [6].

#### Image normalization

T1/T2 MR images were normalized in JPG format using a computational function proposed by D. Catalano [7]. The normalization parameters mean (108.0) and variance (2400.0) were obtained from an array of T1/T2 sagittal MRI images from [8]. This dataset contains an anonymized array of clinical MRI studies of 515 patients with symptomatic back pain. The same normalization can be applied to reduce the dependence on acquisition parameters when calculating time sequences.

#### Data clustering

The better the separation of training data, the more reliable the recognition. Therefore, before training the network, the separability of the dataset is checked to reduce introducing noise. For this purpose, a self-organizing Kohonen map is used. The images for object separation study are represented as sequences of pHash codes [9]. A pHash is a “fingerprint” of a multimedia file derived from various characteristics of its contents. Each image is collapsed to a size of 32×32 pixels and a binary code in the form of a string is computed. An array of these strings is fed to the input of the self-organizing map.

#### Neural network architecture

**Figure 2.**
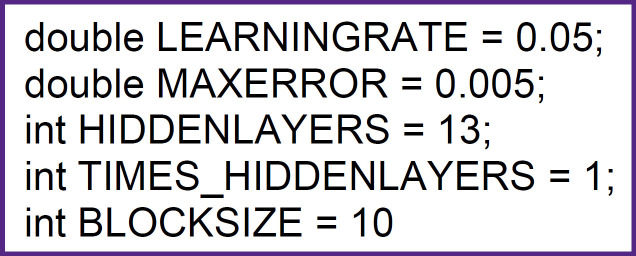
The configuration of the network to reproduce the results obtained

A multilayer perceptron with back propagation training was chosen as the neural network architecture. Two equal competitors Neuroph were chosen for practical implementation [10] and DL4J [11]. A comparison between them did not show a significant superiority of one package over the other [12]. In order to select the best network by minimizing the training error, not one but several networks are trained, i.e., the committee principle is used. The Figure 2 shows the configuration of one network to reproduce the results obtained.

## Results and Examples

### Indications of the drug’s effect at the start of treatment. Arcela marker

A multi-year MRI study of the spine at 3–6 month intervals on the same machine was performed to determine the sequence of action. Bisphosphonates, letrozole and the CDK 4/6 inhibitor palbociclib were used from the beginning.

**Figure 3.**
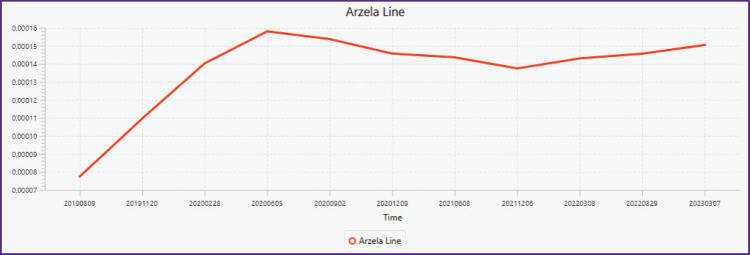
Change the complexity as treatment progressed Measurements performed on Th6 T1 MRI in the sagittal plane over 4 years at 3–6-month intervals on the same machine

Example (Figure 3) describes the change in complexity as treatment progresses, indicating a rapid positive response of Arcela operator values and showing its role as a marker of therapy. Disease stabilization occurred approximately 10 months after surgery to remove the cancerous tumor and initiation of drug treatment.

### Indications for the effect of the drug at the start of treatment. Caldera marker

**Figure 4.**
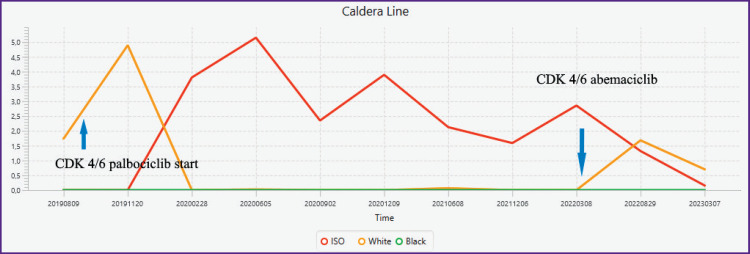
Caldera change as treatment progressed Measurements were performed on Th6 T2 MRI in the sagittal plane for 4 years at 3–6-month intervals on the same instrument. Arrows indicate the time of onset of action of the respective CDK 4/6 inhibitor

As an illustration, we present one of the interesting results (Figure 4). The result was obtained by plotting the time sequence of Caldera values over several years on sagittal T2 MRI, when therapy (due to marked neutropenia with the CDK 4/6 inhibitor palbociclib) was changed to the CDK 4/6 inhibitor abemaciclib.

When switching to abemaciclib, the white caldera (White) reappears, indicating drug activity. As with palbociclib, there is a further decrease in the white caldera (White). The graph also shows that overall, the response to the inhibitor has decreased, and if one roughly considers that the response is proportional to the volume of cancer cells, on the one hand they still persist, on the other hand they have more than halved.

### An additional marker of drug action. Contrast marker

**Figure 5.**
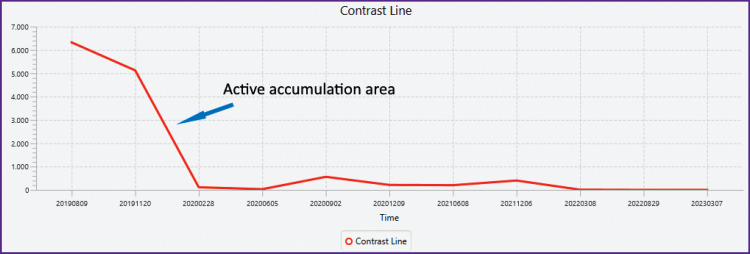
Change in contrast accumulation as influenced by treatment Measurements were performed on T1 sagittal + contrast Th6 MRI for 4 years at 3–6-month intervals on the same machine

Figure 5 shows the curve of change in contrast agent accumulation.

If we compare with the previous curves, the increase in complexity, for example, does not end with the cessation of accumulation and is delayed for another six months. On the other hand, accumulation is almost weakly related to the formation and change of calderas.

### Monitoring data for network training. Example of a sagittal T2 image

**Figure 6.**
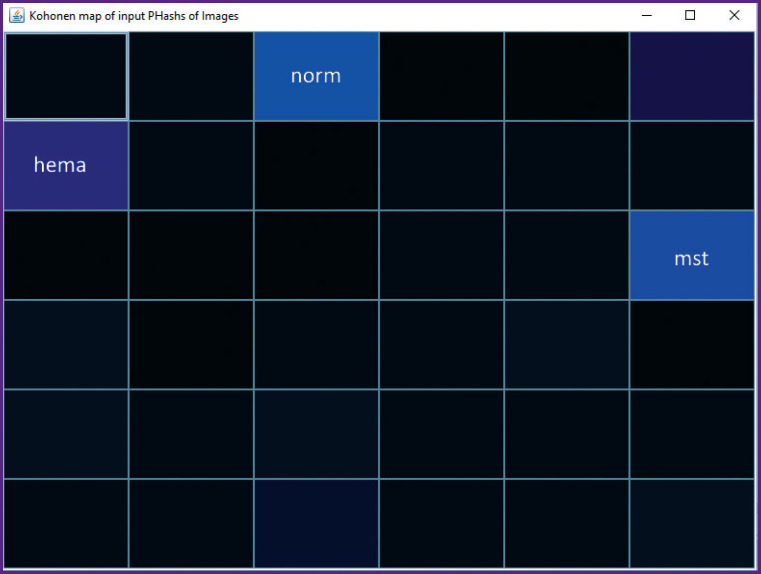
Kohonen map

The training sequence of the partitioning example contains three types of objects: norm — normal bone tissue, mst — metastasis, hema — hemangioma. Therefore, three groups should be expected in the map (Figure 6), which is what is observed with slight deviations and a hint of the 4^th^ group.

Thus, the necessary (but not sufficient, more work needs to be done in this direction) condition for the training sequence is satisfied.

### Example of recognizing metastases on a T2 sagittal image

**Figure 7.**
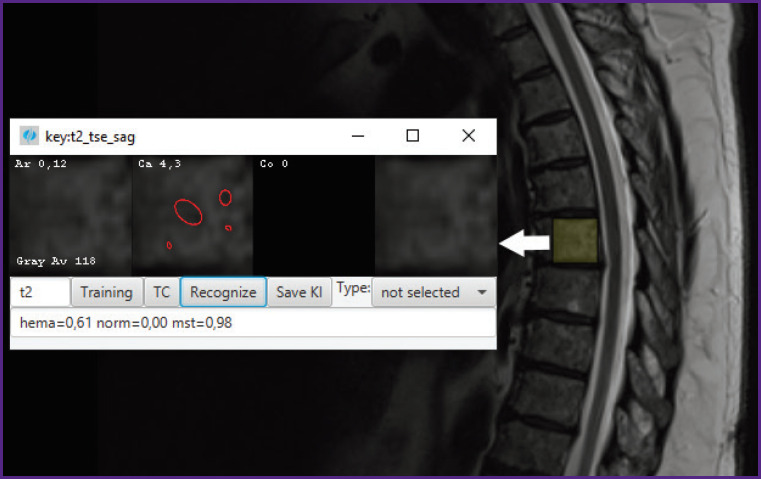
Recognition of a metastatic lesion on Th6 by sagittal T2- weighted MRI: mst probability — 0.98

To illustrate the work, we present an example of recognizing a metastasis in the Th6 vertebra from sagittal T2 data (Figure 7). The result also contains an estimate of Caldera (Ca 4.3%), Arcela score (Ar 0.12, rather low value) and finally probabilities: normal tissue (norm=0), hemangioma (hema=0.61 — unlikely), metastasis (mst=0.98). This gives a clue for analyzing and tracking the time sequence.

### Example of recognizing metastases on a T1 sagittal image and joint interpretation with T2

**Figure 8.**
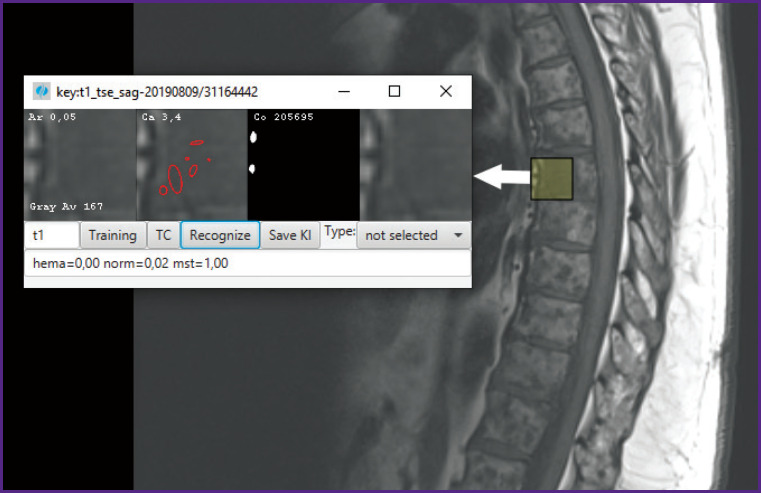
Recognition of a metastatic lesion on Th6 by sagittal data T1-weighted MRI: mst probability — 1.00

To illustrate the work, we also present an example of recognizing a metastasis in the Th6 vertebra from T1 sagittal data (Figure 8). The result also contains a Caldera score (Ca 3.4%), Arcela score (Ar 0.05, quite low), contrast accumulation (none) and finally probabilities: normal tissue (norm=0.02), hemangioma (hema=0.00 — unlikely), metastasis (mst=1.00). This gives a clue to analyze and track the time sequence.

Osteolytic metastases usually have varying degrees of increased signal on T2 sequences. On the other hand, sclerosing metastases have hypointense signal on both T1 and T2 images. Osteolytic metastases have hypointense signal on T1 images. In our case, T1 and T2 measurements indicate osteolytic metastasis.

## Discussion

The facts we have drawn from in this work are as follows: at the microscopic level in the osteoclastic variant of breast cancer, the metastatic lesion usually presents as a radially symmetrical object. As treatment progresses, MR images change and there is an increase in bone density if the tumor regresses. The first manifestation of “healing” on osteolytic metastases of breast cancer is found to be a sclerotic ring around the nidus. With the operators used in the study, we were able to track the response to therapy of breast cancer with bone lytic metastases in the sagittal slice as previously in the axial slice. We realize that this is insufficient to draw unequivocal conclusions, so we have tried to show by real-world example the potential of the algorithms and provide recommendations for practical replication to discuss further development. The limitations of the proposed methods are also to be investigated.

## Conclusion

The use of a small software system with many years of data storage without a dedicated data bank, without the use of complex deep learning with Big Data and thus accessible to any radiologist or oncologist is demonstrated. Examples of the use of “Radiomica Applicata” can be seen in reference [13]. The scope of analysis has been extended to sagittal projection MRI, features such as reducing image complexity in chaotic tumor angiogenesis in the vertebra (Arcela), controlling the formation of elliptically symmetric objects in the vertebra (Caldera). The elliptical formations themselves are classified into three types. All this allows for a deeper understanding and further control of individualized patient treatment by quantifying MDA criteria. The classic joint application of T1 and T2 is also demonstrated. The developed markers can be used in further studies to gain additional insights into the tumor status in bone tissue, as exemplified by changes in CDK 4/6 inhibitor, where it can be seen how new sites are activated.
